# Comparison of preservation and transportation protocols for preloaded Descemet membrane endothelial keratoplasty

**DOI:** 10.1136/bjophthalmol-2017-310906

**Published:** 2017-11-13

**Authors:** Vito Romano, Mohit Parekh, Alessandro Ruzza, Colin E Willoughby, Stefano Ferrari, Diego Ponzin, Stephen B Kaye, Hannah J Levis

**Affiliations:** 1 Department of Ophthalmology, St Paul’s Eye Unit, Royal Liverpool University Hospital, Liverpool, UK; 2 Instituto Universitario Fernandez-Vega, Universidad de Oviedo and Fundacion de Investigacion on Oftalmologica, Oviedo, Spain; 3 International Center for Ocular Physiopathology, Veneto Eye Bank Foundation, Venice, Italy; 4 Department of Eye and Vision Science, University of Liverpool Institute of Ageing and Chronic Disease, Liverpool, UK

**Keywords:** cornea, dystrophy, experimental laboratory, treatment surgery

## Abstract

**Background/aims:**

Descemet membrane endothelial keratoplasty (DMEK) preparation is technically demanding and is a limiting factor for uptake of this kind of surgery. Supply methods that simplify the procedure for surgeons are key to increasing uptake. This study compares two different shipping protocols for DMEK.

**Methods:**

An 8.5 mm DMEK graft was punched, marked and loaded for transportation in two different conditions: (A) endothelium trifolded inwards in organ culture conditions (n=7) and (B) endothelium rolled outwards in hypothermic conditions (n=7). Tissues were shipped from Italy to the UK, then analysed for orientation, endothelial cell density, denuded areas, cell mortality, triple viability staining (Hoechst/ethidium homodimer/calcein AM (HEC)), immunolocalisation of ZO-1 and Na/K-ATPase proteins, visualisation of actin filaments using phalloidin and histological analysis using H&E on paraffin-embedded sections.

**Results:**

All tissues clearly showed the mark used for graft orientation. After shipping in condition A, there was an increase in cell mortality of 8.1% and in denuded areas of 22.4%, whereas for condition B there was an increase in cell mortality of 14.2% and in denuded areas of 34.3% after shipping. HEC staining revealed areas of viable cells and apoptotic cells, with large denuded areas found in the periphery for condition B and within folds for condition A.

**Conclusions:**

Prestripped preloaded DMEK grafts retained sufficient viable cells for transplantation, with condition A (endothelium-in) offering the advantage of greater flexibility of use due to a longer shelf-life. HEC analysis provides further detailed information as to the status of DMEK grafts and should be used in future similar studies.

## Introduction

Corneal transplantation is one of the most frequently performed types of transplant procedure. Despite a large potential donor pool, there exists a global shortage of eye donors.^1^ A recent epidemiological study estimated that only one cornea is available for every 70 needed, with some countries having no access to suitable tissue.^2^ In order to overcome this shortage and improve outcomes, there has been a rapid increase in the number of lamellar surgeries, and the advantages of lamellar keratoplasty over full-thickness keratoplasty are now well established.^3^ There is also increasing evidence that Descemet membrane endothelial keratoplasty (DMEK) shows superior outcomes compared with Descemet stripping automated endothelial keratoplasty in terms of visual outcomes and rehabilitation rates.^4^ Uptake of DMEK, however, is relatively low, possibly because of the skill required in graft preparation. Precut, preloaded tissue, therefore, presents several advantages. For the hospital and surgeon there is reduced complexity, time and associated costs; for the patient, there is improved validation and quality control that are difficult to undertake in the surgical theatre; and for the eye bank, it enables the use of one cornea for more than one recipient. It is essential therefore to optimise the preparation and transport of prestripped, preloaded tissue.

Currently, there are only two preloaded DMEK shipping protocols, originating from the USA^5^ and from Italy.^6^ Tran *et al* have described loading the DMEK graft into a delivery device, such as a modified Jones tube, with the endothelium facing outwards. Scrolling of Descemet’s membrane with the endothelium out is the spontaneous form that the tissue takes within a liquid. This, however, makes handling of the tissue difficult and exposes the endothelial cells to potential damage against the wall of the delivery tube during both loading and insertion. In contrast, the Veneto Eye Bank Foundation (FBOV) and colleagues describe methods with endothelium trifolded inwards, which may facilitate insertion of the tissue both within the introducer and also into the eye with less potential damage to the endothelium.^6–8^

The aim of this study was to compare these two contrasting protocols to assess their effect on endothelial cell survival in preloaded DMEK grafts in order to determine the optimal shipping condition. Determining the most appropriate protocol will facilitate further uptake of DMEK surgery and potentially deliver more consistent and improved surgical outcomes. This would enable an eye bank to ensure high-quality tissues are provided to corneal surgeons in numerous remote locations across the world where access may be limited.^9 10^

## Methods

### Ethical statement

Fourteen human cadaveric donor corneoscleral discs were obtained from FBOV, Venice, Italy, following consent from the donor’s next of kin.

### Shipping conditions

**Figure 1:**
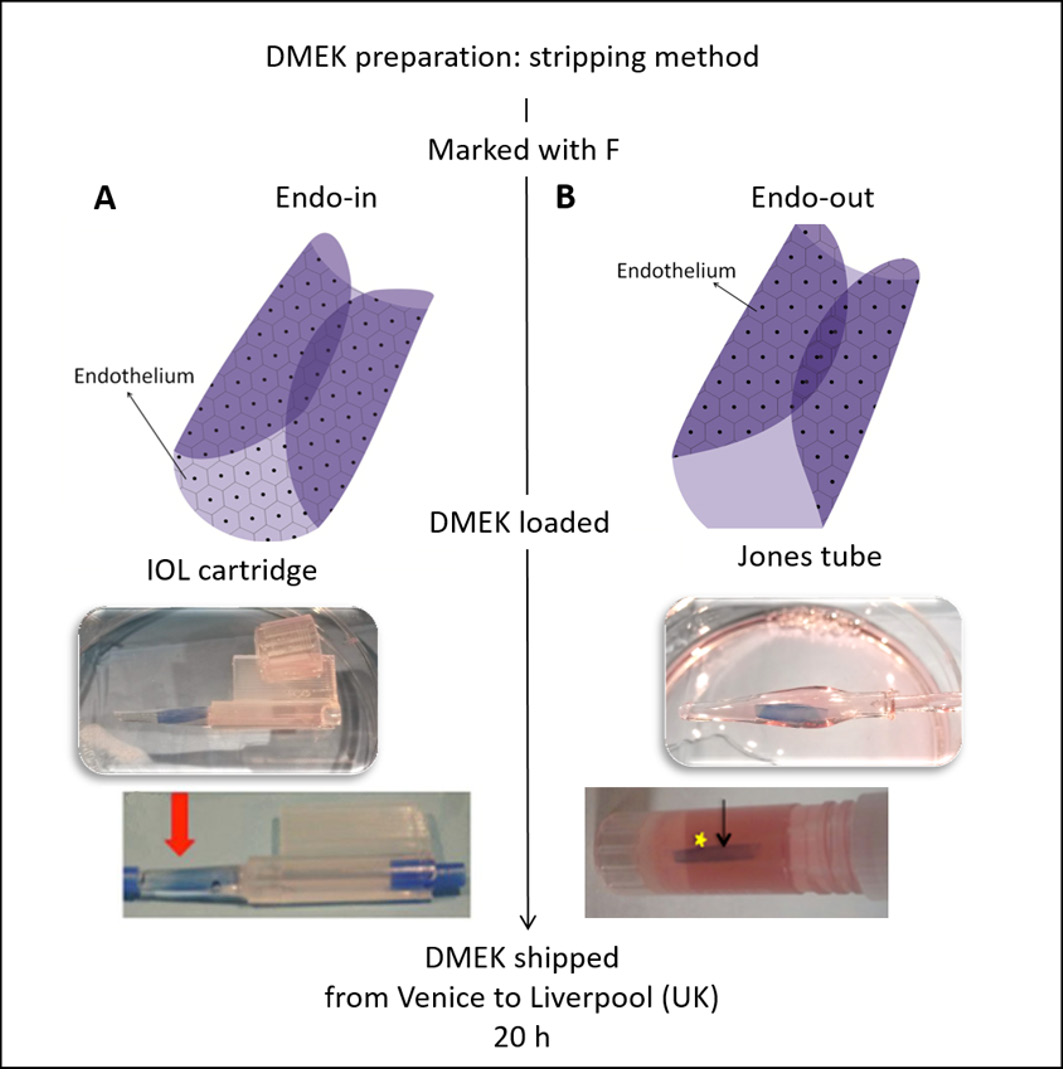
Schematic to depict the two different shipping conditions for Descemet membrane endothelial keratoplasty (DMEK) grafts. (A) Condition A with endothelium-in stored in organ culture medium, and (B) condition B with endothelium-out and stored in Optisol. The arrows on the bottom of the two images indicate the position of the trypan blue-stained scroll within the vessel ready for shipping. The intraocular lens (IOL) cartridge is sealed with stoppers and the Jones tube loaded into an Eppendorf tube.

Condition A was endothelium-flapped inwards and tissue preserved in organ culture medium supplemented with 6% dextran T500 (transport medium) and shipped at room temperature (RT) (figure 1A). For condition B, the endothelium was scrolled outwards in a modified Jones tube preserved in hypothermic medium (Optisol-GS, Bausch & Lomb, Bridgewater, NJ, USA) at 4°C during transportation (figure 1B).

### Preparation of donor tissues in the eye bank for shipment

#### Stripping method

The corneoscleral discs (n=14) were centred on a vacuum punch base with a vacuum created using a syringe. A 9.5 mm trephine (Moria, Antony, France) was used to make a superficial cut using a gentle tapping method. The endothelium was stained with trypan blue (0.4%) for approximately 20 s to determine the area of the cut. Excess peripheral membrane was removed using 120 mm acute forceps (e.janach, Como, Italy). The endothelium was kept moist during the entire procedure using transport media. The membrane was slightly lifted and scored using a cleavage hook around the entire circumference of the cut to ensure minimal tearing of the DMEK tissue while stripping. The DMEK tissue was stripped using a four-quadrant method leaving a hinge behind. Using a skin biopsy punch, a stromal hole was created to mark the endothelium (Descemet’s membrane side) with ‘F’ using a skin marker to aid orientation. Maintaining the correct orientation of the donor graft during DMEK surgery is crucial. If the graft orientation is incorrect on attachment to the corneal stromal surface, primary graft failure may occur. The DMEK tissue was replaced back on the posterior stroma. The tissue was inverted and the stromal piece (punch site) was removed to mark ‘F’ on the Descemet’s membrane, the stromal piece was replaced back, and the corneal tissue was reinverted and fixed again on the vacuum base for loading.

#### Loading method of endothelium-in preloaded DMEK preserved in transport medium (condition A)

As described previously,^6^ the prestripped membrane (9.5 mm) was retrephined with an 8.5 mm punch (Moria) and the excess periphery was removed, maintaining the endothelial side face up (n=7). The membrane was folded manually from two opposite ends using acute forceps, touching the Descemet’s side to avoid endothelial damage. Only a small drop of liquid was added to the membrane to maintain tissue moisture throughout without allowing opening of the membrane, as this may change the orientation. The membrane was carefully moved from the tissue base to a 2.2 intraocular lens (IOL) cartridge (Viscoject, Wolfhalden, Switzerland) using a contact lens as a carrier to maintain the architecture and orientation of the membrane, as previously described.^8^ The membrane was pulled inside the funnel using Grieshaber Revolution DSP 25 G end-grasping forceps (Alcon, Fort Worth, Texas, USA). A small amount of transport medium (<0.5 mL) was used to fill the funnel of the IOL cartridge for preservation of the DMEK membrane. The IOL cartridge was sealed with a rubber plug at both the funnel and the rear exit. The entire unit was sealed with in-house modified rubber clamps and preserved in a sterile phial with transport media. The preloaded tissues were shipped at RT to the University of Liverpool, UK, for further analysis (figure 1A).

#### Loading method of endothelium-out preloaded DMEK preserved in Optisol-GS (condition B)

As described earlier by Parekh *et al*,^6^ following stripping of the Descemet’s membrane (DM), the grafts were freed from the corneal hinge and immediately placed onto a Petri dish containing phosphate buffered saline (PBS; n=7). The graft, as expected, rolled with the endothelium outwards. Using a modified Jones tube and a syringe filled with Optisol-GS, the graft was aspirated inside the modified Jones tube. The entire unit was placed in an Eppendorf tube (2 mL) filled with Optisol-GS and shipped in hypothermic conditions (on wet ice) to the University of Liverpool, UK, for further analysis (figure 1B).

### Tissue analysis with light microscopy

The tissues were analysed before preloading at FBOV and then after the shipment to Liverpool, UK (n=14). The endothelial layer was examined at FBOV using an inverted microscope (Primovert; Zeiss, Jena, Germany) for endothelial cell number, denuded areas, cell mortality and tissue integrity, and presence of ‘F’ orientation mark. The cells were counted using a 10×10 eyepiece reticule and the images were captured at 100× magnification. In Liverpool, the tissues were released from their shipping vessels using end-grasping forceps and analysed. The membranes were stained with trypan blue for 20 s and washed with PBS before further analysis.

### Histological staining

After shipping, two tissue samples from each condition were fixed in 4% paraformaldehyde (PFA), placed on an automated processor overnight and embedded in paraffin prior to sectioning and histological staining. H&E staining was performed on sections to determine any macro changes in DM thickness and the presence of a continuous endothelial layer. Tissue thickness was measured using Fiji image analysis software.

### Tissue fixation and preparation for immunostaining

Samples from each condition were unfolded and placed on a glass slide with endothelium face up. The tissues were fixed in 4% PFA at RT for 30 min. Three tissues were halved and analysed for presence of ZO-1 (n=3), Na+/K+ATPase (n=3) and phalloidin (n=1). The cells were permeabilised with 0.1% Triton X-100 in PBS for 30 min. After blocking with 10% normal goat serum, the tissues were incubated overnight at 4°C with primary antibodies (table 1). The samples were incubated with goat antimouse/rabbit Alexa Fluor 488 secondary antibody in PBS for 2 hours at RT in the dark. For phalloidin staining, tissue was incubated for 2 hours at RT in the dark. Cells were counterstained with 4’,6-diamidino-2-phenylindole, dilactate (DAPI) for 10 min at RT. After each step, the cells were washed three times with PBS and mounted with coverslips and hard set mounting medium. Cells were examined with an LSM 800 confocal microscope (Zeiss).

**Table 1:** Details of antibody and stain concentrations

Antibody/stain	Supplier	Concentration
Zonula Occludens-1 (ZO-1) (mouse)	Thermo Fisher Scientific (40–2200)	2.5 µg/mL
Na+K+ATPase (rabbit)	Santa Cruz (sc-71638)	5 µg/mL
Goat antirabbit Alexa Fluor 488 secondary	Thermo Fisher Scientific (A11008)	4 µg/mL
Goat antimouse Alexa Fluor 488 secondary	Thermo Fisher Scientific (A11001)	4 µg/mL
Alexa Fluor 488 phalloidin	Thermo Fisher Scientific (A12379)	2 units/mL
DAPI dilactate	Thermo Fisher Scientific (D3571)	300 nM

DAPI, 4’,6-diamidino-2-phenylindole.

### Endothelial cell triple staining viability assay

Whole DMEK tissue was triple-stained to determine the viability and coverage of endothelial cells after transport using a Hoechst/ethidium homodimer/calcein AM (HEC) combination as previously described by Pipparelli *et al*.^11^ Cells that are not actively converting calcein but whose membranes are not yet permeable to EthD-1 will stain with Hoechst alone, which we have termed here as intermediate cells. The DMEK tissues were washed with PBS prior to the assay to remove or dilute serum esterase activity generally present in serum-supplemented growth media. Hoechst 33 342 (H; 3 µg/mL, Thermo Fisher Scientific, Rochester, New York, USA), ethidium homodimer EthD-1 (E; 0.8 µM) and calcein AM (C; 0.4 µM) (LIVE/DEAD Viability/Cytotoxicity Kit, Thermo Fisher Scientific) were added to PBS. Approximately 200 µL of the final solution was added to the DMEK tissue on a glass slide and incubated at RT in the dark for 45 min. Relaxing radial cuts were made at three points to obtain a flat mount and tissue protected with a coverslip without mounting medium. HEC staining was viewed with an LSM 800 confocal microscope. A tile scan was performed using a 5× objective and reconstructed using ZEN processing software to produce an image of staining across the whole surface of the graft. Trainable Weka segmentation on Fiji was used to analyse the percentage area covered by viable cells, intermediate cells and denuded areas as previously described by Jardine *et al*.^12^ Briefly, the images were converted into 16-bit, black and white, and the pixel intensity adjusted for contrast. The trainable segmentation plugin was activated and classes assigned to live, intermediate and denuded areas. The software was then trained to detect these areas and then train classifier selected; retraining was performed if necessary. A final segmentation was produced, and using the histogram feature on Fiji a pixel count was acquired to derive a percentage for each category. General instructions for use of this plugin can be found at http://Fiji.sc/Trainable_Segmentation_Plugin.

### Statistical analysis

Data are expressed as mean±SD and paired data were analysed using paired Wilcoxon test for non-parametric data. P value <0.05 was considered significant.

## Results

### Donor characteristics

Average donor age was 72.5 (±6.8) years with 11 men and three women. The average postmortem interval between death and retrieval was 9.8 (±3.9) hours.

### Tissue preparation

Time to prepare (second punch, folding and loading) tissue in condition A was approximately 20 min compared with condition B which took only 5 min, which is similar to previously published results.^7^ Peeling success was 100% (n=14). Time of shipment between Italy and UK was approximately 20 hours. All the tissues were analysed between 48 and 72 hours after preloading.

### Initial tissue analysis after shipping

**Figure 2:**
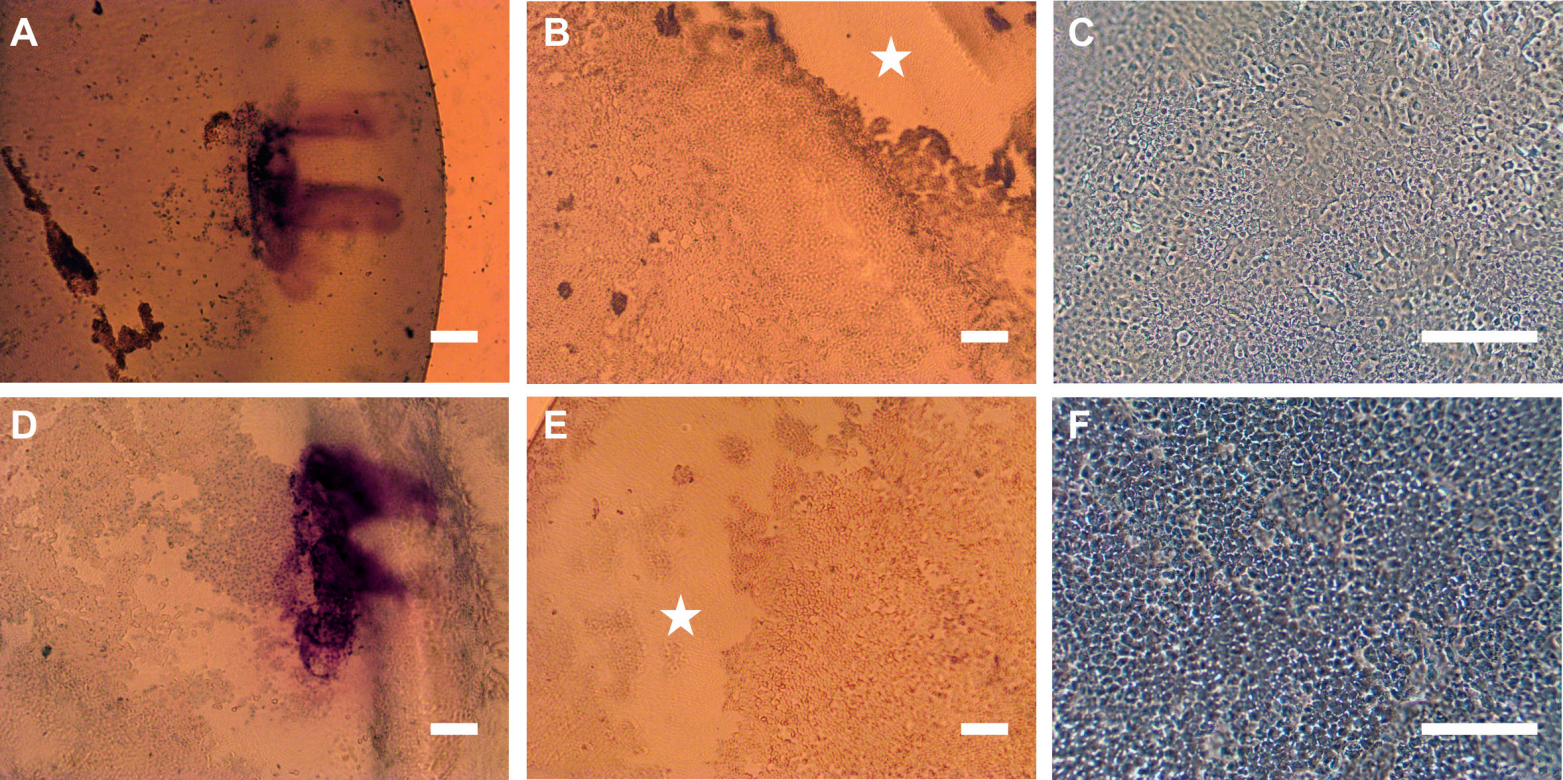
Representative images of the F mark (A,D), denuded areas in the periphery of grafts (stars; B,E) and central endothelial cells (C,F) after shipping of preloaded Descemet membrane endothelial keratoplasty tissues in condition A (A–C) and condition B (D–F). Scale bars: 250 µm.

All but one of the tissues were clearly visible in their delivery device. One preloaded graft required restaining with trypan blue to aid visualisation. The ‘F’ mark to indicate orientation was prominent on all the samples; however, there was evidence of cell loss surrounding marks (figure 2A,D). For condition A, the endothelial cell density was 1928.57 (±111.27) cells/mm^2^ before shipping and on second analysis there was an increase in cell mortality, as indicated by trypan blue staining, from 1.93% (±3.59) to 10.0% (±5.77) and denuded areas from 0% to 22.43% (±13.89). Meanwhile for condition B, the endothelial cell density was 1785.71 (±146.39) cells/mm^2^, with cell mortality increased from 6.57% (±3.91) to 20.7% (±30.72) and denuded areas from 0% to 34.28% (±15.11) after shipping. There was no statistically significant difference in cell mortality and denuded areas between the two conditions for these parameters. Figure 2 shows the far periphery of the endothelium (figure 2B,E) and the central endothelium after shipping of the preloaded DMEK grafts (figure 2C,F).

### Histological examination of DMEK tissue

**Figure 3:**
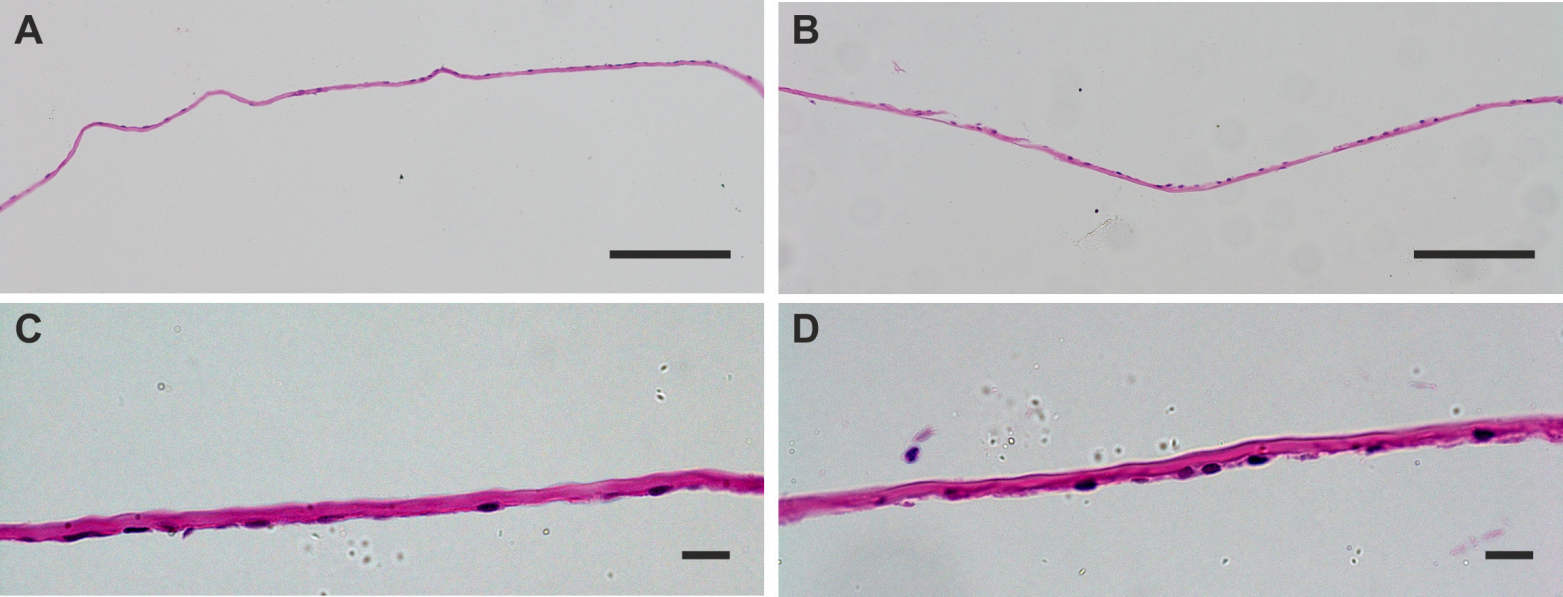
H&E-stained sections of Descemet membrane endothelial keratoplasty (DMEK) tissue after shipping in condition A (A,C) and condition B (B,D). Note that the thickness of the DMEK grafts are comparable, and in both conditions there is a visible monolayer of endothelial cell nuclei stained with haematoxylin. Scale bars: A and B: 100 µm; C and D: 10 µm.

H&E staining revealed a monolayer of cells, and tissue thickness was on average 3.61 µm (±0.64) for condition A and 4.59 µm (±0.89) for condition B (figure 3). This indicated no macroscopic changes in DM thickness and distribution of endothelial cells after shipping.

### Expression of functional proteins in endothelial cells on DMEK tissue

**Figure 4:**
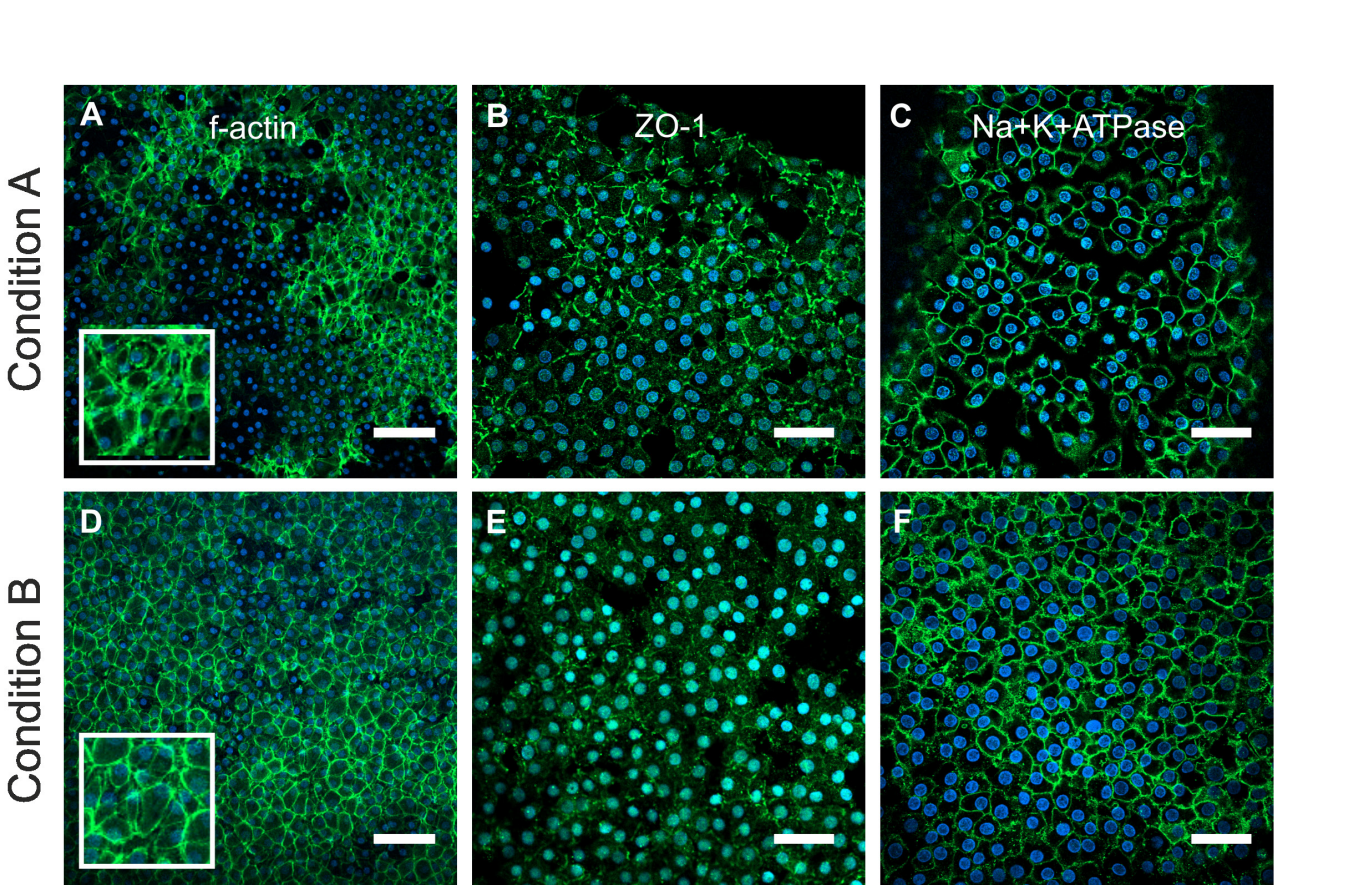
Representative images showing expression of f-actin (A,D; phalloidin-stained, green), ZO-1 (B,E; green) and Na+/K+ATPase (C,F; green) in endothelial cells counterstained with 4’,6-diamidino-2-phenylindole (DAPI) (blue) after shipping in condition A (A–C) or condition B (D–F). Insets in A and D show higher magnification of phalloidin-stained areas. Scale bars: A and D: 100 µm; B, C, E and F: 50 µm.

The expression of ZO-1, Na+/K+ATPase and f-actin was analysed in whole-mount DMEK tissue (figure 4). Phalloidin staining revealed heterogeneous f-actin distribution throughout the endothelial layer in condition A (figure 4A), with some areas showing intense cortical f-actin staining and condensed foci at the Y-junctions with neighbouring cells. The distribution of f-actin in condition B (figure 4D) cells was generally homogeneous with prominent staining of cortical f-actin bundles running parallel to cell borders. ZO-1 expression (figure 4B,E) was similar between shipping conditions with a discontinuous expression observed at the cell borders. Na+/K+ATPase expression was also comparable between conditions, localised to the cell membranes (figure 4C,F).

### HEC triple viability staining of DMEK tissue

**Figure 5:**
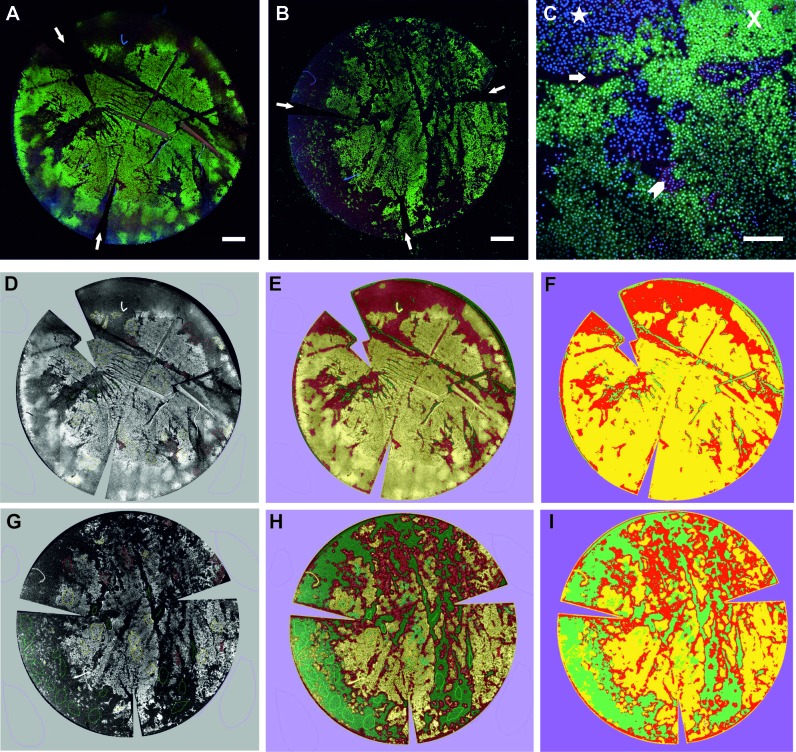
Confocal images of HEC staining of grafts after shipping in condition A (A) and B (B). Note the relaxing incisions to enable flat mounting of grafts (A,B; white arrows). Calcein viable cells (green and X) and Hoechst intermediate cells (blue and star) can be seen in whole mount images. Denuded areas (arrow) and ethidium homodimer non-viable cells (red and chevron) can be seen at higher magnification (C). Weka segmentation processing of a graft shipped in condition A (D–F) and condition B (G–I). Confocal images of HEC-stained DMEK grafts were processed using Fiji into 16-bit images (D,G), then training was performed using Weka segmentation to define the areas of live (yellow), intermediate (red) and denuded areas (green; E,H). The final segmentation image result was then reproduced in three colours that were quantified with Fiji histogram tool (F,I). Scale bars: A and B: 1 mm; C: 200 µm. DMEK, Descemet membrane endothelial keratoplasty; HEC, Hoechst/ethidium homodimer/calcein AM.

HEC was used to identify distinct areas of viable, non-viable, intermediate cell-covered areas as well as denuded areas across the whole tissue in both conditions (figure 5A–C). Using the Weka trainable segmentation plugin on Fiji, the percentage areas covered by viable, intermediate cells and denuded areas could be quantified (figure 5D–I and table 2). There appeared to be a higher number of viable cells present on the graft after shipping in condition A compared with B, and large denuded areas were visible in the condition B graft.

**Table 2:** Percentage area of Descemet membrane endothelial keratoplasty tissue covered by viable and intermediate cells and denuded areas

Condition	Viable (%)	Intermediate (%)	Denuded areas (%)
A	70.1	24.4	5.5
B	37.8	30.9	31.3

## Discussion

DMEK requires great skills in both the surgical delivery of the graft to the posterior cornea and also the preparation of tissue. There is a necessity to develop methods to increase the uptake of the DMEK procedure by surgeons worldwide. Preloaded grafts can reduce the amount of tissue wastage and reduce surgery time as the process is performed by highly trained and experienced eye bank staff. In addition to conventional endothelium-out preloaded DMEK grafts, preloaded DMEK grafts have been produced with the endothelium folded in (the opposite way to its natural scrolling tendency), in an attempt to decrease endothelial cell loss due to contact with delivery device and to increase the ease of opening of tissue inside the anterior chamber.^13^

This study shows that both preloaded DMEK protocols retained sufficient viable cells for transplantation. Denuded areas, cell mortality and live/dead/apoptotic analyses suggest a trend towards increased viability with preloaded DMEK endothelium-in (condition A), although this result is limited by the small sample size. It is worth noting that the size of the shipping vessel may play a role in the degree of cell loss, in particular in the endothelial-out scrolls as they will contact the vessel walls during the shipping process. This is a preloaded method with the graft shipped ready for injection into the anterior chamber, however, so there is a limit to the size/shape of vessel that can be used as the graft needs to be easily injected from the shipping vessel. It was interesting to note the high levels of intermediate cells in both conditions. These cells were not actively converting the calcein AM to calcein, but their membranes were not yet permeable to ethidium. This population may be missed using conventional live/dead viability staining, and so this test should be included in the future to give a clearer picture of cell health in similar studies or in studies of endothelial cell health related to postmortem time from death to enucleation, for example. The conventional method of imaging and recording endothelial density does not provide as much information as triple staining of the whole graft surface and delineation of denuded areas. Immunocytochemical staining of grafts using ZO-1 and Na+/K+ATPase antibodies to ensure tight junctions are intact and surface pumps are functional suggested that the majority of cells still remaining on the surface of the graft are functional.

The F mark to enable orientation of the graft was prominent in all the tissues; however, it was noted that there was occasionally an area of surrounding damage at the sites where the mark was present with either high amounts of mortality or denuded areas. It is worth noting that the F mark is usually the point where the microforceps contact the graft while inserting into the IOL cartridge or during implantation. Therefore, denuded areas at that point could be expected and may not be solely due to the method of producing the F mark. The F mark in condition B grafts resulted in some cell mortality but not in denuded areas as there was no direct contact with the forceps. We have previously noted that the F mark induces around 0.5%–1% cell mortality of the whole tissue.^8^ This level of cell loss may be acceptable as marking the graft helps to reduce intraocular manipulation of the graft, reduce surgery time, and therefore reduce the rate of primary graft failure. Others have tried alternative methods such as three semicircular marks at the periphery of the graft made using a 1 mm trephine,^14^ or making a single peripheral triangular mark with an area of 0.44 mm^2^.^15^ The physical removal of tissue obviously leads to the loss of a proportion of tissue and endothelial cells and could lead to decreased graft attachment at the periphery, but is perhaps a more durable solution rather than marking with a letter, although in our study all F marks were clearly visible after shipping. At this stage, the choice of marking method may be purely based on personal preference of the surgeon.

The aim of this study was to compare two different preloaded DMEK protocols in their entirety. Therefore, in addition to the comparison of shipping/graft delivery device, our study compared two different types of storage medium used in the different protocols. European eye banks store corneas in organ culture medium at 31°C after retrieval from cadavers and before transplantation. The tissues are then transferred into dextran-based medium for shipping to reduce the oedema and related corneal thickness.^16^ In contrast, in the USA, the tissues are preserved throughout the storage period in hypothermic medium containing dextran and maintained at 4°C.^17^ The advantage of the organ culture-stored corneas is that they have a longer shelf-life, approximately 30 days compared with 14 days for hypothermic stored tissue, which gives increased flexibility for their usage. Our previous study has shown that excising a DMEK graft that has been stored in dextran-based medium is easier (100% success) compared with excision from organ culture media without dextran (76% success).^18^ Standardised preparation methods may further reduce graft wastage. In our earlier studies we have shown that preloading a DMEK graft in an IOL cartridge does not lead to significant cell loss (<10%) when preserved in dextran-based medium at RT for 4 days. Preserving a preloaded DMEK graft in tissue culture medium, however, leads to higher cell loss (approximately 35%).^6 7^ This present study shows that prestripped, preloaded DMEK tissues shipped in organ culture medium plus dextran are comparative, and superior in some aspects, in suitability for transplant when compared with Optisol-stored grafts. The longer shelf-life and shipping temperature (RT) required for organ culture-stored grafts mean that this method may be more suitable for shipping to remote locations, expanding access to tissue worldwide.

Preloaded donor tissue will minimise surgical time and risks associated with preparation of DMEK, eliminating possible complications in the operating room. This will also reduce wastage of human corneal tissues, potentially providing a more standardised tissue. The prepared loading of the donor tissue into the single use delivery device can be advantageous. The increased costs related to precutting and preloading the tissue are balanced by the reduction in surgical time and instrumentation required to prepare the tissue. Regardless of the conditions and preparation or delivery methods, preloaded DMEK appears to be the future of lamellar surgery and may help less experienced surgeons to easily uptake this challenging surgery.
